# Leptin attenuates heat stress-induced mitoxyperilysis in porcine Sertoli cells by suppressing the mtDNA-cGAS-STING axis

**DOI:** 10.1186/s40104-026-01489-6

**Published:** 2026-09-06

**Authors:** Ming Guo, Jinyang Zhang, Shanshan Gu, Yawen Gao, Zhendong Zhu, Tao Zhang, Hongzhao Lu, Jun Luo, Wenxian Zeng

**Affiliations:** 1 College of Animal Science and Technology, Northwest A&F University, Yangling, Shaanxi 712100 China; 2 School of Biological Science and Engineering, Shaanxi University of Technology, Hanzhong, Shaanxi 723001 China; 3 College of Animal Science and Technology, Qingdao Agricultural University, Qingdao, 266109 China

**Keywords:** cGAS-STING, Heat stress, Leptin, Mitoxyperilysis, mtDNA leakage

## Abstract

**Background:**

Mammalian spermatogenesis is highly temperature-sensitive and relies critically on the protective function of Sertoli cells. Although heat stress is known to affect Sertoli cell fate, it remains unclear whether it triggers mitoxyperilysis and by what mechanism. Leptin is a multifunctional hormone that regulates energy metabolism and is critical for mitochondrial integrity across various cell types. However, whether leptin safeguards Sertoli cells from heat stress‑induced mitoxyperilysis remains unclear.

**Results:**

In this study, the results demonstrated that heat stress induced mitochondrial oxidative damage, leading to mitochondrial DNA (mtDNA) leakage and metabolic reprogramming. Mechanistically, heat stress compromised mitochondrial membrane integrity by promoting aberrant mPTP opening, triggering BAX/BAK oligomerization, and facilitating VDAC1 oligomerization, collectively leading to mtDNA leakage into the cytosol. The cytosolic mtDNA activated the cGAS-STING signaling, subsequently triggering TBK1-IRF3 and NF-κB signaling cascades, which drove inflammatory cytokine production and ultimately culminated in mitoxyperilysis. Importantly, leptin safeguarded mitochondrial integrity, effectively prevented mtDNA release and suppressed cGAS-STING activation, thereby attenuating mitoxyperilysis.

**Conclusions:**

Collectively, these findings suggest that leptin may serve as a critical guardian of mitochondrial integrity that protects Sertoli cells from heat stress-induced mitoxyperilysis, highlighting its potential to support future therapeutic strategies for heat stress-related male reproductive dysfunction.

**Supplementary Information:**

The online version contains supplementary material available at https://doi.org/10.1186/s40104-026-01489-6.

## Introduction

Global warming-induced heat stress adversely affects livestock reproductive performance. In vivo studies indicate that sustained exposure to high temperature impairs spermatogenesis and diminishes semen quality in boars [1, 2]. Acute heat stress is widely used as an in vitro model to investigate the molecular events triggered by thermal insult, including oxidative stress, mitochondrial dysfunction, and inflammatory signaling. Understanding these early cellular responses is essential for elucidating the mechanisms underlying heat stress-associated male infertility [3–5].

Testicular tissue exhibits a highly specialized and complex architecture, comprising multiple cell types that are spatially organized. Sertoli cells, the sole somatic cells within the seminiferous tubules, establish and maintain the specialized microenvironment necessary for spermatogenesis by forming the blood-testis barrier, regulating immune homeostasis, and mediating paracrine signaling [6]. Studies have demonstrated that heat stress induces mitochondrial reactive oxygen species (mtROS) accumulation in Sertoli cells, resulting in decreased mitochondrial membrane potential, impaired ATP synthesis, and suppressed activity of key tricarboxylic acid cycle enzymes [7–9]. These alterations disrupt cellular energy metabolism and redox homeostasis, leading to metabolic reprogramming [8].

Mitochondrial membrane disruption and the consequent leakage of mitochondrial constituents represent a pivotal event that couples cellular stress to the activation of inflammatory pathways. The specific molecular triggers, however, are highly context-dependent [10–13]. In cardiomyocytes, heat stress and doxorubicin elicit pathological mitochondrial permeability transition pore (mPTP) opening and the release of mtDNA [12, 13]. In melanocytes, H₂O₂ induces mtDNA leakage via voltage-dependent anion channel (VDAC1) oligomerization [11], whereas in colon cancer cells, hypoxia-reoxygenation promotes BAX/BAK-dependent outer membrane permeabilization, facilitating mtDNA efflux [10]. Thus, although membrane breach is a common terminal event, upstream mechanisms are dictated by the specific stressor and cell type. It remains unclear whether heat stress induces mtDNA leakage in Sertoli cells.

Cytosolic mtDNA is sensed by cGAS, which in turn activates the cGAS-STING signaling pathway [12, 14]. Subsequent STING activation triggers downstream TBK1-IRF3 and IκBα-NF-κB signaling, which drive the synthesis and secretion of pro-inflammatory factors and promote inflammatory response [15]. Li and colleagues reported that exposure to titanium dioxide nanoparticles induced excessive reactive oxygen species (ROS) production and DNA damage in mouse Sertoli cells, leading to upregulation of cGAS and STING [16]. However, whether heat stress triggers mtDNA leakage in Sertoli cells and engages the cGAS-STING pathway remains to be elucidated.

Recently, Wang et al. [17] reported that under lipopolysaccharide stimulation combined with carbon starvation, macrophages exhibited prolonged mitochondrial-plasma membrane contact, which led to local lipid peroxidation and membrane blebbing, ultimately causing plasma membrane rupture and cell death, termed mitoxyperilysis. This novel form of cell death occurs independently of caspases, gasdermins, MLKL, and ferroptosis regulators. *Bax*/*Bak* double-knockout significantly reduced mitoxyperilysis, indicating that BAX/BAK are essential mediators of mitoxyperilysis [17]. However, it remains unclear whether BAX/BAK oligomerization induces mtDNA leakage to drive mitoxyperilysis.

Leptin, an adipocyte-derived hormone, enhances mitochondrial resistance to apoptosis in neurons [18], attenuates hypoxia/reoxygenation-induced activation of programmed cell death in rat H9c2 cells [19] and inhibits the activation of cGAS-STING pathway and protects cells from oxidative stress-induced damage [20]. Furthermore, leptin suppresses the nuclear factor kappa B (NF-κB) signaling pathway and reduces the secretion of pro-inflammatory cytokines, such as interleukin-6 (IL-6) and tumor necrosis factor-α (TNF-α) [21]. A previous study has demonstrated that exogenous leptin peptide treatment improves testicular function under hyperthermic conditions [22]. Interestingly, leptin receptor is notably expressed in Sertoli cells [23, 24]. Nevertheless, whether leptin inhibits cGAS-STING activation in Sertoli cells, thereby attenuating heat stress-induced inflammatory responses, remains unclear. Likewise, it is unknown whether leptin protects Sertoli cells from mitoxyperilysis.

Therefore, this study aims to investigate whether leptin protects against acute heat stress in Sertoli cells by maintaining mitochondrial integrity, reducing mtDNA leakage, and thereby suppressing the cGAS-STING pathway and mitigating mitoxyperilysis. Understanding this mechanism is crucial for developing strategies to prevent heat stress-induced male infertility.

## Materials and methods

### Experimental design

All Sertoli cells were seeded simultaneously and allowed to adhere overnight. The total culture duration after the initiation of treatments was kept identical (27.5 h) across all four groups. The experimental design was as follows: Control group (Ctrl): cells were cultured at 37 °C for 27.5 h. Leptin-treated group (Lep_ctrl): cells were cultured under 37 °C for 3.5 h, then supplemented with 6 nmol/L leptin and incubated for an additional 24 h (total 27.5 h). Heat stress group (HS): cells were exposed to 43 °C for 1.5 h, then returned to 37 °C for a recovery period of 2 h, followed by further culture for 24 h without any leptin treatment (total 27.5 h). The heat stress protocol was established according to previous studies in Sertoli cells [3–5]. Heat stress plus leptin group (Lep_hs): cells were exposed to 43 °C for 1.5 h, allowed to recover for 2 h, then treated with 6 nmol/L leptin for 24 h (total 27.5 h).

### Sertoli cell collection

Sertoli cells were isolated from the testes of 7-day-old Landrace piglets obtained from Shaanxi Shunxin American Line Breeding Pig Farm, using a combination of enzymatic digestion and differential adhesion, based on previously established protocols with slight modifications [25]. The comprehensive experimental procedures are described in detail in Text S1 (Additional file 2). 

### Immunofluorescence staining

Cells were fixed in 4% paraformaldehyde and permeabilized with 0.25% Triton X-100 in PBS. Subsequently, the cells were blocked with SuperBlock™ blocking buffer (Thermo Fisher Scientific, USA) and incubated overnight at 4 °C with primary antibodies (Table S1). The following day, after washing with PBS, secondary antibody incubation and DAPI staining were performed. Finally, images were acquired using a fluorescence microscope (Olympus, China).

### Detection of intracellular ROS

Intracellular total ROS levels were measured using a ROS Assay Kit (Beyotime, China). Briefly, cells were incubated with DCFH-DA at 37 °C for 20 min in the dark and then analyzed by flow cytometry. ROS levels were quantified as mean fluorescence intensity.

### Detection of mitochondrial ROS

The level of mtROS was assessed by a Mitochondrial Superoxide Detection Kit (Beyotime, China). Cells were incubated with MitoSOX at 37 °C for 30 min in the dark and then analyzed by flow cytometry. The level of mtROS was quantified as mean fluorescence intensity.

### Measurement of mitochondrial membrane potential

Mitochondrial membrane potential was assessed using an enhanced mitochondrial membrane potential assay kit (Beyotime, China). Cells were incubated with 1 × JC-1 working solution for 30 min in the dark. Fluorescence intensity was measured using a microplate reader, and the ratio of red to green fluorescence was calculated as an indicator of mitochondrial membrane potential.

### Determination of intracellular ATP content

ATP content was quantified using an ATP Assay Kit (Beyotime, China), following the manufacturer’s instructions. Briefly, cells were lysed in ATP-releasing buffer and centrifuged at 12,000 × *g* for 5 min at 4 °C. The supernatant was mixed with a luciferin-luciferase reagent, and luminescence was measured using a microplate reader (BioTek, USA). ATP levels were normalized to total protein and expressed as nmol/mg protein.

### Measurement of the enzymatic activity of citrate synthase

Citrate synthase activity was determined using a Citrate Synthase Activity Assay Kit (Solarbio, China), according to the manufacturer’s instructions. Briefly, cell lysate was combined with Acetyl-CoA and oxaloacetate, and the formation of 5-thio-2-nitrobenzoic acid was monitored at 412 nm. Citrate synthase activity was normalized to total protein content and expressed as U/mg protein.

### Glycolytic enzyme activity and metabolite assays

To evaluate the activities of key rate-limiting enzymes in glycolysis and the levels of glycolytic products, the activities of hexokinase (Solarbio, China), phosphofructokinase (Beyotime, China), pyruvate kinase M (Beyotime, China) and lactate dehydrogenase (Beyotime, China) were measured using commercial assay kits. Lactate levels were determined using a lactate assay kit (Nanjing Jiancheng Bioengineering Institute, China), and pyruvate levels were measured using a pyruvate assay kit (Beyotime, China). All procedures were performed according to the manufacturer’s instructions, and detailed protocols are described in Text S2.

### CCK-8 assay

Briefly, the Cell Counting Kit-8 (CCK-8) reagent (Beyotime, China) was diluted 1:10, incubated with the cells for 30 min, and the absorbance was then measured at 450 nm.

### qPCR assay

Total RNA was extracted from cells in different treatment groups using TRIzol™ reagent. Complementary DNA was synthesized from 1 μg of total RNA using the PrimeScript RT Reagent Kit (DIYIBio, China). qPCR was performed on an IQ5 Real-Time PCR System (Shanghai Hongshi Medical Technology, China) using 2 × Hieff PCR Master Mix (No Dye) (YEASEN, China) in a final reaction volume of 20 μL. All reactions were run in triplicate. β-actin was used as an internal reference gene for normalization. Relative mRNA expression levels were calculated using the 2^−ΔΔCt^ method. The primer sequences used are listed in Table S2.

### Western blot assay

Total protein was extracted from Sertoli cells using RIPA lysis buffer supplemented with protease and phosphatase inhibitors. Protein concentration was quantified using the BCA Protein Assay Kit (DIYIBio, China). Equal quantities of protein were separated by SDS-PAGE and transferred onto PVDF membranes. Membranes were blocked with 5% non-fat milk in TBST (10 mmol/L Tris-HCl, 150 mmol/L NaCl, and 0.1% Tween-20, pH 7.4) for 1 h at room temperature, followed by incubation with primary antibodies overnight at 4 °C. After washing with TBST, membranes were incubated with HRP-conjugated secondary antibodies for 1 h at room temperature. Protein bands were detected using an ECL detection system. β-Actin was used as a loading control. Antibody details are summarized in Table S1.

### Assessment of mitochondrial permeability transition pore (mPTP) opening

The mPTP was detected by a Mitochondrial Permeability Transition Pore Assay Kit (Beyotime, China). Briefly, cells were washed with PBS and incubated with Calcein AM (2.5 μmol/L) plus CoCl_2_ (10 mmol/L) at 37 °C for 30 min. After washing, cells were analyzed by flow cytometry. A decrease in Calcein AM fluorescence intensity was considered indicative of increased mPTP opening.

### Detection of intracellular calcium ion content

Intracellular calcium levels were evaluated using the Fluo-4 AM probe (Beyotime, China). Briefly, Sertoli cells were incubated with 5 μmol/L Fluo-4 AM in culture medium for 30 min at 37 °C in the dark. Cells were then analyzed by flow cytometry.

### Mitochondrial DNA stress response assay

To investigate whether heat stress triggers mtDNA leakage, we used fluorescence colocalization imaging and absolute quantitative real-time PCR. Cells were subjected to heat stress and/or leptin treatment. Subsequently, cells were co-stained with MitoTracker Deep Red FM (Cell Signaling Technology, USA) and the mtDNA-specific probe MitoDNA Green 530 (AAT Bioquest, USA). Confocal images were acquired using a laser-scanning confocal microscope (Leica STELLARIS 5, Germany). Simultaneously, colocalization analysis of mitochondrial and mtDNA signals was conducted to evaluate the extent of mtDNA release. DNA extracted from cytoplasmic fractions was analyzed by absolute qPCR using a standard curve method with mtDNA-specific primers to quantify mtDNA abundance. Comprehensive experimental protocols are described in Text S3.

### Enzyme-linked immunosorbent assay (ELISA)

The content of IL-6 or IL-1α was measured in cells from various treatment groups. Porcine IL-6 (Elabscience, E-EL-P3008) and IL-1α (Elabscience, E-EL-P1437) ELISA kits were used to measure IL-6 and IL-1α, respectively, according to the manufacturer’s instructions.

### Assessment of mitoxyperilysis

To assess whether mitoxyperilysis occurred, fluorescence colocalization microscopy combined with flow cytometry was performed. After treatment, cells were co-stained with the mitochondrial dye MitoTracker Deep Red FM and the plasma-membrane probe DiO (YEASEN, China). Briefly, MitoTracker Deep Red FM was diluted 1:2,000 and incubated with cells at 37 °C for 20 min. Subsequently, DiO was diluted 1:1,000 and incubated at 37 °C for 15 min. Finally, nuclei were counterstained with Hoechst at room temperature for 10 min. Confocal images were acquired using a laser-scanning confocal microscope (Leica STELLARIS 5, Germany).

To evaluate the level of intracellular lipid peroxidation, the fluorescent probe BODIPY 581/591 C11 was used. The probe was diluted at a ratio of 1:2,000 and incubated with the cells for 30 min at room temperature. Subsequently, the cells were centrifuged at 450 × *g* for 5 min at 4 °C, and the mean fluorescence intensity of each treatment group was detected by flow cytometry.

Flow cytometry was used to measure mean fluorescence intensity of PI-positive cells and to quantify the proportion of PI-positive cells. For PI staining, PI was diluted 1:100 and cells were incubated on ice for 30 min, followed by immediate flow cytometric analysis.

### Statistical analysis

SPSS 22.0 software was used for statistical analysis. GraphPad Prism 9.0 was used for data visualization. All experiments were performed at least three times independently. One-way analysis of variance (ANOVA) with Tukey's multiple comparisons test was used to analyze independent sample datasets. Different lowercase letters above bars indicate significant differences between groups. Data were presented as the mean ± SD, and differences were considered statistically significant at *P* < 0.05.

## Results

### Leptin alleviates acute heat stress-induced mitochondrial oxidative damage and metabolic imbalance in Sertoli cells

**Fig. 1:**
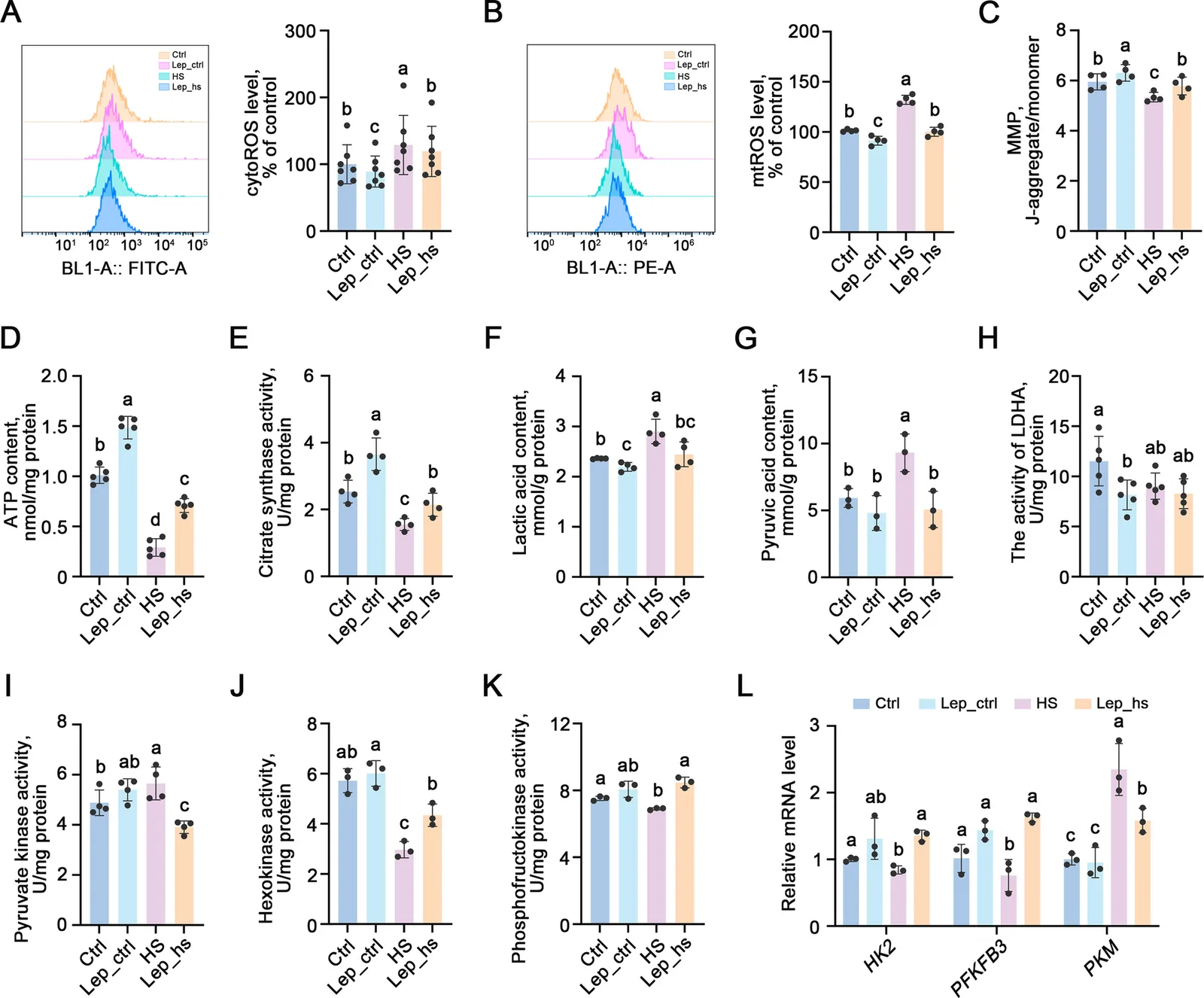
Leptin alleviates heat stress-induced mitochondrial oxidative damage and metabolic imbalance in Sertoli cells. Sertoli cells were divided into four groups: Ctrl, Lep_ctrl, HS, and Lep_hs. For clarity, Ctrl and Lep_ctrl are presented together under basal conditions, whereas HS and Lep_hs are presented together under heat stress conditions. **A** Cytoplasmic ROS (cytoROS) levels were measured using DCFH-DA staining and quantified as mean fluorescence intensity, expressed as a percentage of the control group. **B** Mitochondrial ROS (mtROS) levels were assessed using MitoSOX Red and presented as a percentage of the control. **C** Mitochondrial membrane potential was determined by JC-1 staining, expressed as the fluorescence ratio of JC-1 aggregates to monomers. **D** Intracellular ATP content was measured and normalized to total protein, nmol/mg protein. **E** Citrate synthase activity was assessed and expressed as U/mg protein. **F** and **G** Lactate (**F**) and pyruvate (**G**) levels were measured and normalized to total protein, mmol/g protein. **H** The activity of lactate dehydrogenase (LDHA) was measured and normalized to total protein, U/mg protein. **I**–**K** Activities of glycolytic rate-limiting enzymes, including pyruvate kinase (**I**), hexokinase (**J**), and phosphofructokinase (**K**). **L** Relative mRNA levels of *HK2*, *PFKFB3*, and *PKM*, determined by qPCR, β-actin was used as the reference gene. Data are presented as the mean ± SD of at least three independent experiments (*n* ≥ 3). Different lowercase letters above the bars indicate significant differences among groups (*P* < 0.05)

Immunostaining for the specific marker SOX9 confirmed that isolated Sertoli cells reached a purity of 95%, which was sufficient for subsequent experiments (Fig. S1A). We subsequently characterized the cellular response to heat stress by evaluating redox homeostasis and mitochondrial function. Heat stress significantly elevated ROS levels (Fig. 1A). Given that mitochondria are a primary source of ROS, we measured the level of mtROS and found it was markedly elevated under heat stress (Fig. 1B), suggesting that mitochondria are early targets of heat stress-induced oxidative damage.

We next assessed the impact of this oxidative damage on mitochondrial function. Heat stress reduced the mitochondrial membrane potential (ΔΨm) and cellular ATP content (Fig. 1C and D). Furthermore, enzyme activity assays revealed that heat stress significantly inhibited the activity of citrate synthase, a rate-limiting enzyme of the tricarboxylic acid cycle (Fig. 1E). Thus, we next examined whether heat stress triggers a compensatory shift in energy metabolism. Heat stress led to a substantial accumulation of lactate and pyruvate (Fig. 1F and G). Notably, lactate dehydrogenase activity remained unaltered (Fig. 1H), suggesting a redirection of metabolism rather than a global upregulation of glycolysis. Glycolytic enzyme assays revealed that heat stress increased pyruvate kinase activity (Fig. 1I) but decreased hexokinase and phosphofructokinase activities (Fig. 1J and K). Corresponding changes were observed in the mRNA levels of glycolytic genes (Fig. 1L).

To identify the optimal leptin concentration for mitigating heat stress, cell viability under heat stress was evaluated with different concentrations of leptin (3, 6, 9, and 12 nmol/L). The results showed that leptin at the concentration of 6 nmol/L significantly enhanced cell viability (Fig. S1B). This concentration was therefore selected for all subsequent experiments.

Notably, leptin treatment significantly mitigated heat-induced accumulation of both ROS and mtROS (Fig. 1A and B), partially restored ΔΨm and ATP levels, and increased activity of citrate synthase (Fig. 1C–E). Furthermore, leptin improved metabolic homeostasis by reducing the accumulation of lactate and pyruvate (Fig. 1F and G), and regulating the activity and expression of key glycolytic enzymes (Fig. 1H–L). Collectively, these results suggest that leptin alleviates heat stress-induced mitochondrial dysfunction and metabolic imbalance in Sertoli cells.

### Leptin stabilizes mitochondrial membranes against heat stress-induced damage and prevents mtDNA leakage

**Fig. 2:**
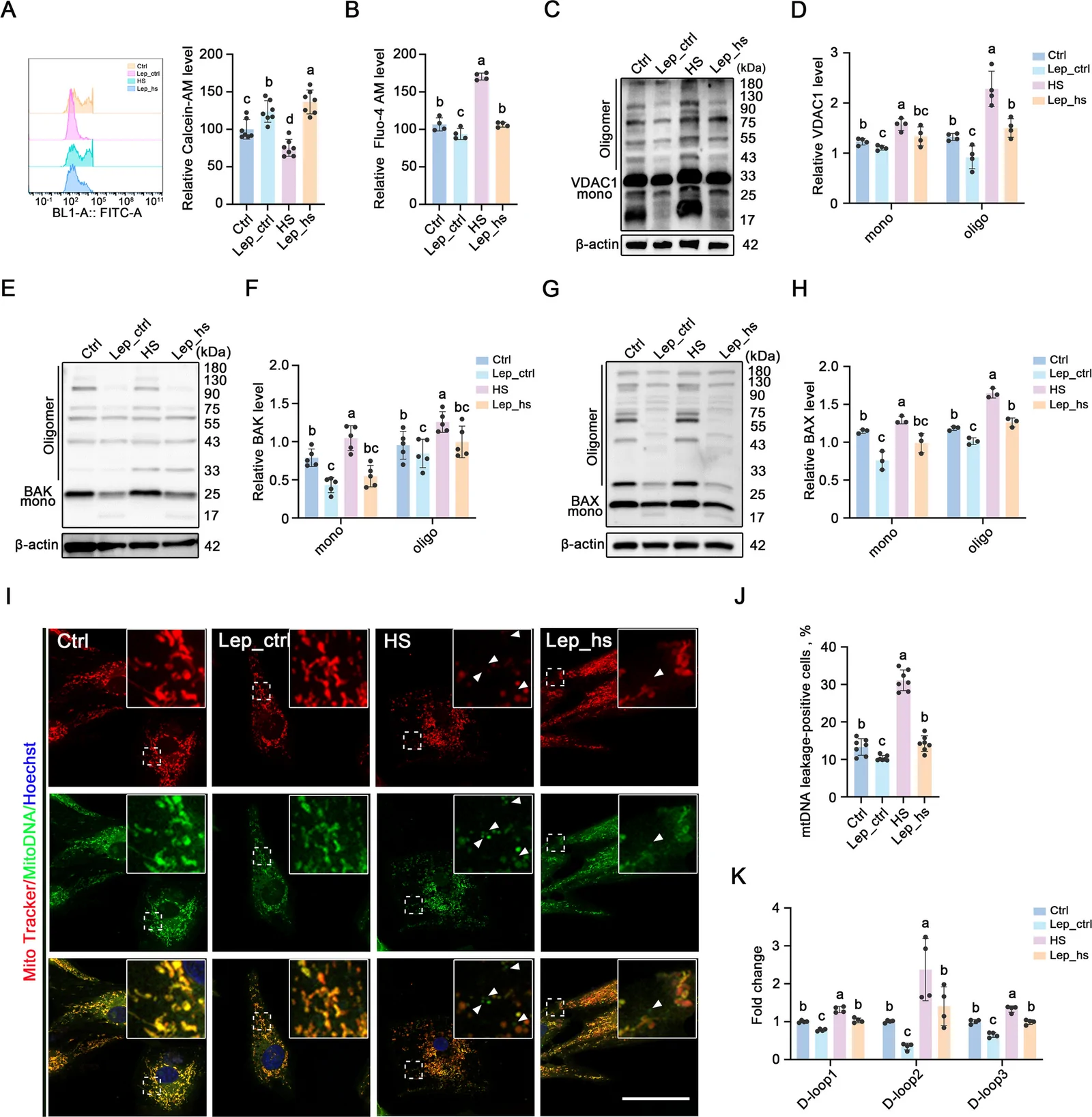
Leptin stabilizes mitochondrial membranes against heat stress-induced damage and prevents mtDNA leakage. **A** Flow cytometry was performed to measure mPTP opening using Calcein-AM, and the mean fluorescence intensity was quantified and normalized to the control. A higher mean fluorescence intensity reflects a lower extent of mPTP opening. **B** Flow cytometry was performed to measure intracellular Ca^2^⁺ levels using Fluo-4 AM, and the mean fluorescence intensity was quantified and normalized to the control. **C** Western blot detected the level of VDAC1 monomer and oligomer levels. **D** Quantification of VDAC1 monomer and oligomer levels, normalized to β-actin. **E** and **F** Representative Western blot image (**E**) and quantification of BAK monomer and oligomer levels (**F**). **G** and **H** Representative Western blot image (**G**) and quantification of BAX monomer and oligomer levels (**H**). **I** Representative confocal images assessing mtDNA leakage: Mitochondria (MitoTracker, red), mtDNA (MitoDNA Green, green) and nuclei (Hoechst, blue). Dashed boxes indicate enlarged regions. Scale bar = 50 μm. **J** Quantification of mtDNA-positive cells in different groups. **K** Relative cytosolic mtDNA content in Sertoli cells in different groups. Data are presented as the mean ± SD of at least three independent experiments (*n* ≥ 3). Different lowercase letters above the bars indicate significant differences among groups (*P* < 0.05)

To investigate whether mitochondrial dysfunction triggers mtDNA release, we measured cytosolic mtDNA levels after heat stress. As mtDNA release requires permeabilization of mitochondrial membranes, we first assessed the opening of mPTP, a large channel between the inner and outer mitochondrial membranes that regulates the influx and efflux of substances, including mtDNA [26]. Our results demonstrated that leptin attenuated heat stress-induced mPTP opening (Fig. 2A) and decreased intracellular calcium levels (Fig. 2B). However, mtDNA release also involves permeabilization of the mitochondrial outer membrane. Therefore, we analyzed mitochondrial outer membrane proteins. Western blot analysis showed that heat stress significantly increased both the expression and oligomerization of VDAC1, which were reversed by leptin treatment (Fig. 2C and D). Furthermore, BAX/BAK are known to form macro-pores in the outer mitochondrial membrane, thereby facilitating the release of macromolecules [10, 26, 27]. Our results revealed that leptin reduced both heat stress-induced expression and oligomerization of BAX and BAK (Fig. 2E–H).

To observe mtDNA release into the cytosol, we labeled mtDNA with the specific probe MitoDNA Green 530 and mitochondria with MitoTracker. We observed that mtDNA was mainly confined to mitochondria in the Ctrl group, whereas heat stress induced partial labeling of mtDNA in the cytosol, indicating mtDNA leakage from mitochondria into the cytoplasm (Fig. 2I). Leptin treatment largely prevented this redistribution, maintaining mtDNA within mitochondria (Fig. 2I). Consistently, heat stress significantly increased the proportion of cells exhibiting mtDNA leakage, while leptin markedly reduced this proportion (Fig. 2J). Absolute quantification of cytosolic mtDNA further confirmed that heat stress significantly elevated D-loop1, D-loop2, and D-loop3 fragment abundance, whereas leptin treatment effectively suppressed these increases (Fig. 2K).

To further determine the pathway responsible for mtDNA release, we performed pharmacological inhibition experiments targeting VDAC1 and BAX/BAK oligomerization. Compared with the heat stress group, treatment with either a VDAC1 oligomerization inhibitor (VBIT-4) or a BAX/BAK oligomerization inhibitor (MSN-50) significantly reduced cytosolic mtDNA levels, as reflected by decreased D-loop1, D-loop2, and D-loop3 fragment abundance (Fig. S2A–C). Notably, inhibition of BAX/BAK oligomerization resulted in a greater reduction in D-loop1, D-loop2, and D-loop3 fragment abundance than inhibition of VDAC1 oligomerization, suggesting that the BAX/BAK-mediated pores may play a more prominent role in heat stress-induced mtDNA release (Fig. S2A–C).

Furthermore, leptin treatment reduced the abundance of D-loop1, D-loop2 and D-loop3 fragments under treatment with either inhibitor alone (Fig. S2A–C). These findings suggest that leptin reduces heat stress-induced mtDNA leakage by limiting mitochondrial membrane permeabilization involving both VDAC1 and BAX/BAK, while additional protective mechanisms may also contribute to its inhibitory effect on mtDNA release.

Overall, these results suggest that heat stress is associated with impaired mitochondrial membrane integrity and increased mtDNA leakage into the cytosol, while leptin effectively preserves membrane stability and restricts mtDNA release.

### Leptin inhibits heat stress‑induced activation of the mtDNA‑cGAS-STING inflammatory pathway by preserving mitochondrial integrity

**Fig. 3:**
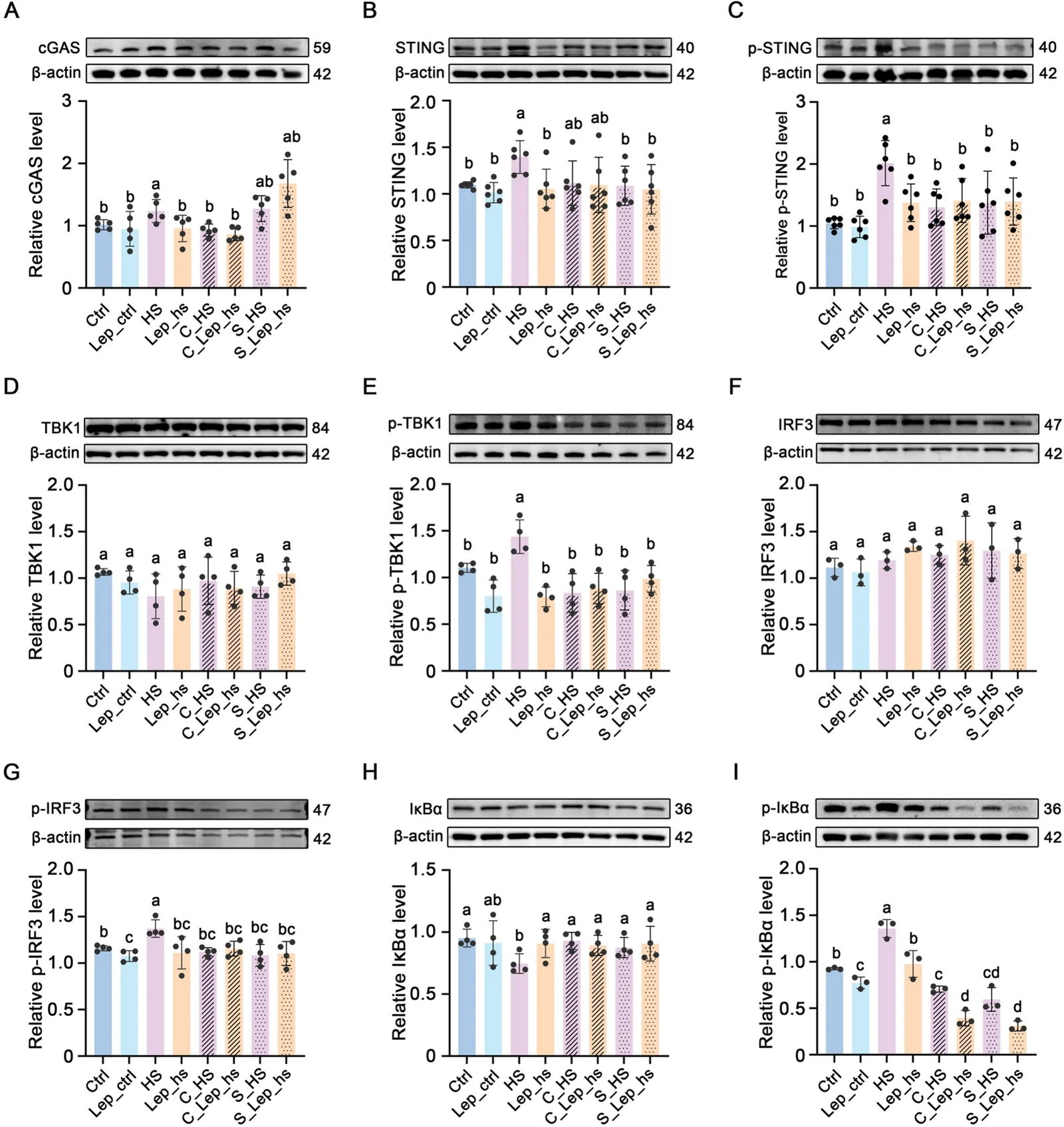
Leptin suppresses heat stress-induced cGAS-STING activation. Eight experimental groups were set up: Ctrl, Lep_ctrl, HS, Lep_hs, C_HS, C_Lep_hs, S_HS, and S_Lep_hs. C represents RU.521 (an inhibitor of cGAS), and S indicates C176 (an inhibitor of STING). Different colors indicate treatment groups, while patterned bars represent heat stress (HS)-combined conditions. **A**–**I** Western blot analysis was used to detect the levels of cGAS (**A**), STING (**B**), p-STING (**C**), TBK1 (**D**), p-TBK1 (**E**), IRF3 (**F**), p-IRF3 (**G**), IκBα (**H**), and p-IκBα (**I**) after treatment with the cGAS inhibitor (RU.521, 10 μmol/L) and the STING inhibitor (C176, 5 μmol/L), with normalization to β-actin. Data are presented as the mean ± SD of at least three independent experiments (*n* ≥ 3). Different lowercase letters above the bars indicate significant differences among groups (*P* < 0.05)

Since leaked mtDNA has been reported to activate the cGAS-STING signaling pathway [28–30], we next examined whether the mtDNA release under heat stress triggers this pathway. Western blot analysis revealed that heat stress significantly increased the expression levels of cGAS, STING and phosphorylated STING (p‑STING) (Fig. 3A–C). To investigate whether mtDNA release is the primary driver of cGAS-STING activation, we employed VBIT‑4 or MSN‑50 to inhibit mtDNA release. Under heat stress conditions, treatment with either VBIT‑4 or MSN‑50 markedly attenuated the upregulation of cGAS and STING, as well as p‑STING (Fig. S2D–F). These findings suggest that heat stress-induced mtDNA leakage may contribute to activation of the cGAS-STING signaling pathway.

**Fig. 4:**
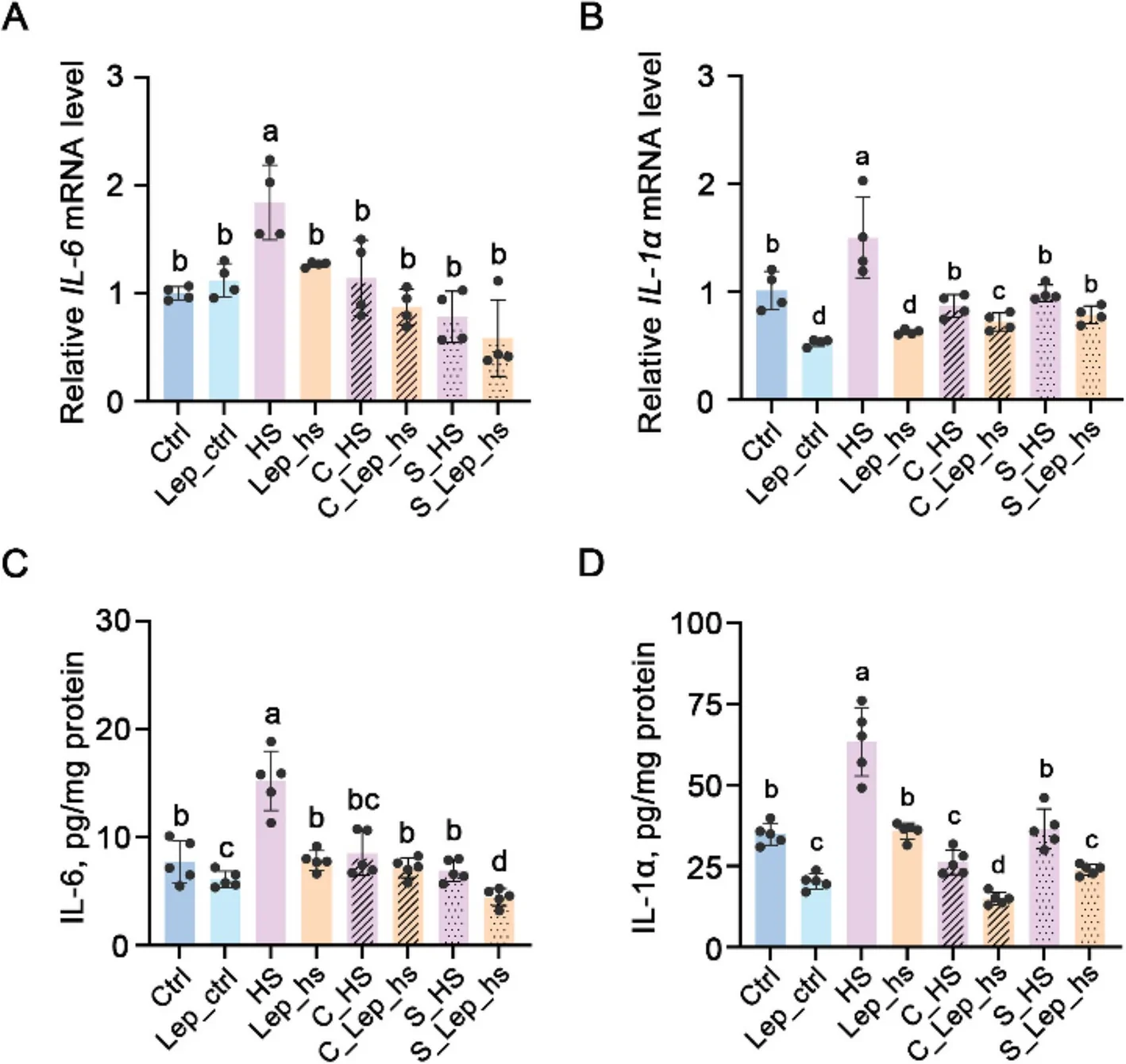
Leptin inhibits cGAS-STING-mediated inflammation under heat stress. **A** and **B** Relative mRNA levels of *IL‑6* (**A**) and *IL‑1α* (**B**) determined by qPCR in different groups. **C** and **D** IL‑6 (**C**) and IL‑1α (**D**) protein levels measured by ELISA and normalized to total protein, pg/mg protein. Data are presented as the mean ± SD of at least three independent experiments (*n* ≥ 3). Different lowercase letters above the bars indicate significant differences among groups (*P* < 0.05)

Given that TBK1-IRF3 phosphorylation is a canonical downstream event following cGAS-STING activation, we next examined this signaling axis. Western blot assays revealed that leptin attenuated heat stress-induced phosphorylation of TBK1 and IRF3 (Fig. 3D–G). The cGAS-STING pathway is known to activate the NF-κB signaling cascade. Western blot analysis showed that leptin inhibited heat stress-induced phosphorylation and degradation of IκBα (Fig. 3H and I). Furthermore, leptin reduced heat stress-induced p65 upregulation and inhibited its nuclear translocation (Fig. S3A and B). Consequently, qPCR and ELISA analyses indicated that leptin attenuated the heat stress-induced upregulation of the pro-inflammatory cytokines IL-6 and IL-1α (Fig. 4A–D).

To determine whether the inflammatory response is triggered by mtDNA leakage via the cGAS-STING axis, we utilized the cGAS inhibitor RU.521 and the STING inhibitor C176. The results showed that under heat stress conditions, treatment with RU.521 or C176 alone markedly reduced the levels of p-TBK1, p-IRF3, p-IκBα, p65 (Fig. 3D–I and Fig. S3A), and the inflammatory cytokines IL-6 and IL-1α (Fig. 4A–D). Moreover, the addition of leptin did not further reduce the levels of these upstream signaling molecules (Fig. 3D–I and Fig. S3A), but it further decreased the protein level of IL‑6 and IL‑1α (Fig. 4C and D).

Taken together, these findings support the involvement of mtDNA leakage and cGAS-STING activation in heat stress-induced inflammatory responses. Leptin effectively alleviates this process, underscoring its potential as a protective agent against heat stress-induced inflammation.

### Leptin attenuates heat stress‑induced mitoxyperilysis by preserving mitochondrial integrity and suppressing cGAS-STING activation

**Fig. 5:**
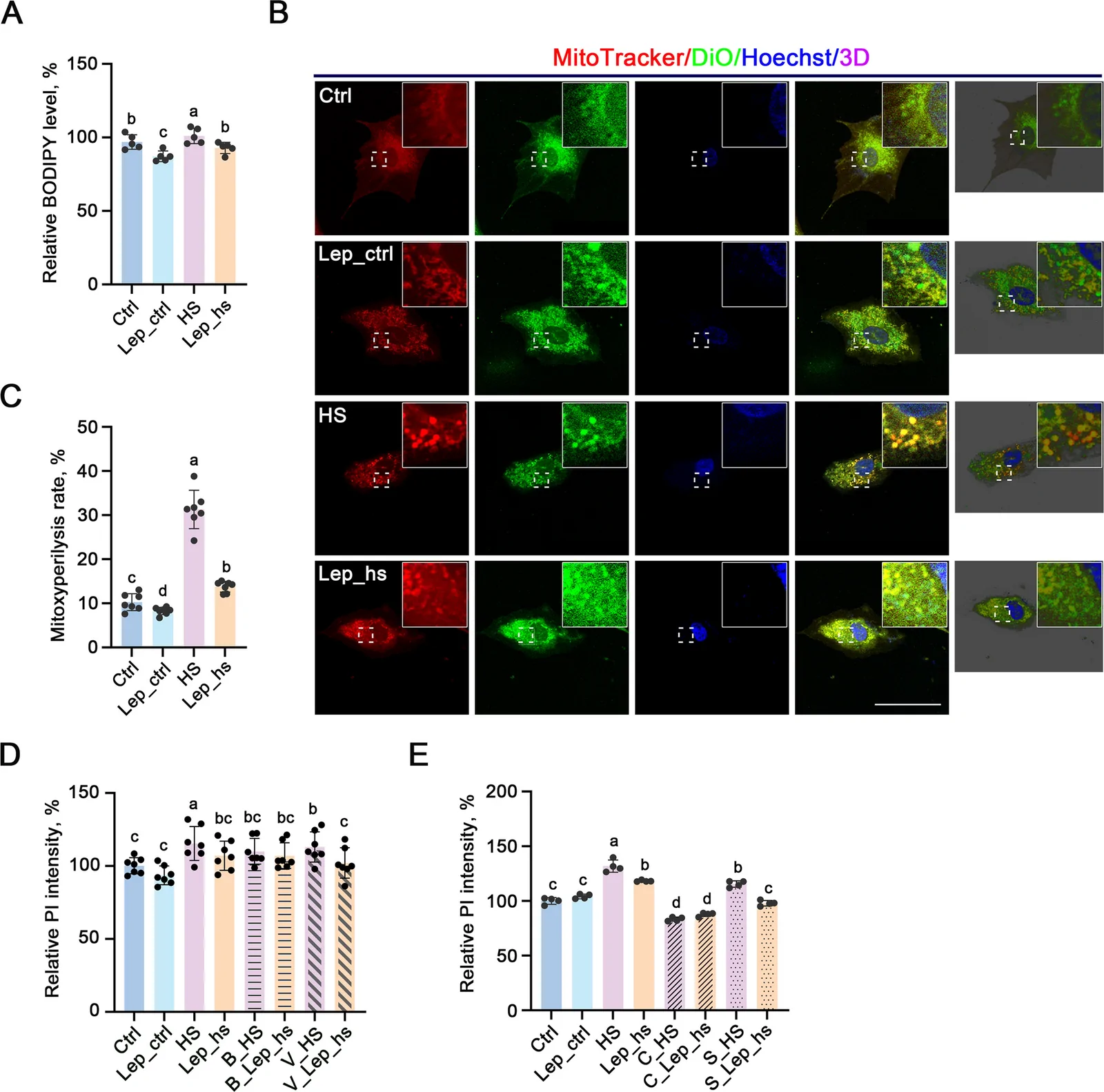
Leptin mitigates heat stress-induced mitoxyperilysis. **A** Flow cytometric analysis of BODIPY 581/591 C11 staining showing the relative BODIPY levels in each group. The mean fluorescence intensity (MFI) was quantified and normalized to the Ctrl group. **B** Cells were co-stained with the plasma membrane dye DiO (green) and the mitochondrial probe MitoTracker (red), with nuclei stained by Hoechst (blue). Yellow signals in the merged images indicate DiO and MitoTracker colocalization, which was used to assess mitochondrial contacts with the plasma membrane/cytoplasmic region. The last column shows a 3D reconstruction of confocal z-stack images acquired by confocal laser scanning microscopy to visualize mitochondrial spatial distribution. Dashed boxes highlight the areas selected for magnification; the upper-right insets show the corresponding high-magnification views. Scale bar = 50 μm. **C** Quantification of the proportion of cells undergoing mitoxyperilysis in different treatment groups. **D** Flow cytometric detection of relative PI fluorescence intensity in heat-stressed Sertoli cells with leptin intervention and pretreatment of BAX/BAK oligomerization inhibitor (MSN-50) or VDAC1 inhibitor (VBIT-4). Eight groups were included: Ctrl, Lep_ctrl, HS, Lep_hs, B_HS, B_Lep_hs, V_HS, and V_Lep_hs. B indicates MSN-50 (an inhibitor of BAX/BAK oligomerization), and V denotes VBIT-4 (an inhibitor of VDAC1 oligomerization). **E** Flow cytometric analysis of relative PI fluorescence intensity in leptin-treated heat-stressed cells after inhibition of cGAS (RU.521) or STING (C176). Data are presented as the mean ± SD of at least three independent experiments (*n* ≥ 3). Different lowercase letters above the bars indicate significant differences among groups (*P* < 0.05)

To investigate whether heat stress induces mitoxyperilysis, we first measured lipid peroxidation using the fluorescent probe BODIPY 581/591 C11. Our result showed that heat stress significantly increased the mean fluorescence intensity of BODIPY 581/591 C11, whereas leptin treatment markedly attenuated this increase (Fig. 5A). Subsequently, we performed confocal live‑cell imaging with the plasma membrane dye DiO and the mitochondrial dye MitoTracker. We observed that MitoTracker foci stably localized near the plasma membrane in the HS group (Fig. 5B). In contrast, MitoTracker foci were predominantly localized in the cytoplasm in the Lep_hs group (Fig. 5B). Consistently, heat stress significantly increased the proportion of cells undergoing mitoxyperilysis, while leptin markedly reduced this proportion (Fig. 5C). Flow cytometry results showed that heat stress significantly increased the proportion of PI‑positive cells, indicating a loss of membrane integrity (Fig. 5D). Leptin treatment markedly reduced the number of PI‑positive cells.

To investigate whether heat stress induces mitoxyperilysis through the mtDNA-cGAS-STING axis, we first inhibited mtDNA release using VBIT-4 and MSN-50. Flow cytometric analysis showed that blocking mtDNA release markedly reduced the percentage of PI-positive cells under heat stress (Fig. 5D). Similarly, pharmacological inhibition of cGAS and STING using RU.521 and C176 also significantly decreased the proportion of PI-positive cells (Fig. 5E). These results indicate that heat stress promotes mitoxyperilysis at least in part via the mtDNA-cGAS-STING axis.

**Fig. 6:**
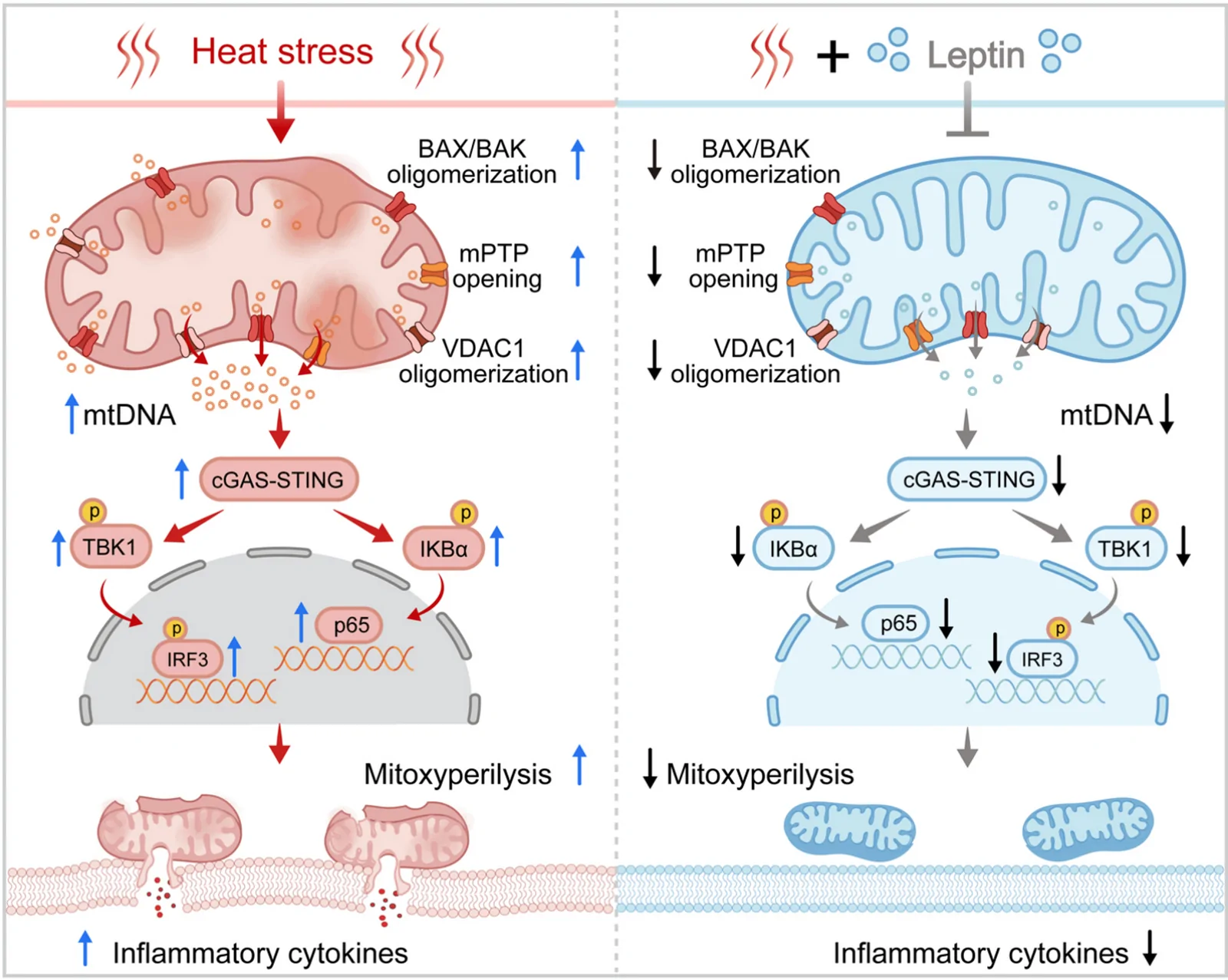
Leptin mitigates heat stress-induced mitoxyperilysis. Heat stress disrupts mitochondrial integrity by increasing mPTP opening, and promoting VDAC1 and BAX/BAK oligomerization, leading to mtDNA leakage into the cytosol. Leaked mtDNA activates the cGAS-STING signaling, triggering TBK1 and IRF3 phosphorylation, as well as IκBα phosphorylation and nuclear translocation of p65, ultimately inducing mitoxyperilysis. Notably, leptin mitigates mitoxyperilysis by preserving mitochondrial membrane integrity, preventing mtDNA leakage into the cytoplasm, and suppressing subsequent cGAS-STING activation. Collectively, leptin ultimately attenuates heat stress-induced mitoxyperilysis

Collectively, these results suggest that heat stress promotes mitoxyperilysis at least in part through the mtDNA-cGAS-STING axis, accompanied by mitochondrial localization near the plasma membrane and reduced membrane integrity, whereas leptin attenuates this process by inhibiting cGAS-STING signaling and contributing to membrane stability (Fig. 6).

## Discussion

Heat stress is a major environmental cause of male infertility, to which Sertoli cells are particularly susceptible. Using a widely adopted acute heat stress model (43 °C for 1.5 h), this study reveals that heat stress compromises mitochondrial membrane integrity by inducing BAX/BAK oligomerization and/or oligomerization of VDAC1, and promoting abnormal mPTP opening, ultimately leading to the leakage of mtDNA into the cytosol. The cytosolic mtDNA subsequently triggers activation of the cGAS-STING signaling pathway, resulting in mitoxyperilysis. Importantly, leptin counteracts this damage cascade through a multifaceted way that preserves mitochondrial membrane integrity, thereby effectively intercepting the heat stress-induced injury pathway. These findings not only delineate the key molecular pathway through which heat stress induces mitoxyperilysis but also provide a mechanistic basis for further investigating the role of leptin in heat stress-associated male reproductive disorders.

### Leptin mitigates heat stress-induced mtDNA leakage

In this study, we demonstrated that heat stress triggered mtDNA leakage into the cytosol, as evidenced by a significant increase in cytosolic D-loop fragment abundance and the accumulation of distinct mtDNA foci in the cytoplasm. Notably, leptin treatment effectively prevented this leakage by preserving mitochondrial integrity through multiple ways. Firstly, it protected the inner mitochondrial membrane. We observed that heat stress induced aberrant mPTP opening, which aligned with previous reports in cardiomyocytes [13]. Notably, our results further demonstrated that leptin treatment effectively suppressed this heat-induced response, thereby safeguarding the structural integrity of the mitochondrial inner membrane. Secondly, leptin secured the outer mitochondrial membrane. We found that leptin inhibited heat stress‑induced BAX/BAK oligomerization, a process that formed pores in the outer mitochondrial membrane and facilitated mtDNA release into the cytosol [27, 31, 32]. Consistently, Jing et al. [10] reported that BAX/BAK activation promoted mtDNA release under hypoxic conditions, and knockdown of these genes markedly reduced cytosolic mtDNA levels in colon cancer cells. In the current study, we found that leptin significantly downregulated the levels of BAX and BAK, which were consistent with previous reports in placental cells under hypoxic conditions [33]. Thirdly, leptin maintained the homeostasis of membrane channel protein. We found that heat stress promoted VDAC1 oligomerization, which was in line with observations in hydrogen peroxide‑treated epidermal melanocytes [11] and embryonic fibroblasts [34]. Remarkably, leptin inhibited this VDAC1 oligomerization. A recent structural and biochemical study demonstrated that apoptotic stimuli induce VDAC1 oligomerization, which promotes exposure of the VDAC1 N-terminal α-helix. The exposed N-terminus can bind and neutralize the anti-apoptotic protein Bcl-xL, thereby facilitating BAX/BAK-mediated mitochondrial outer membrane permeabilization [35]. This model supports the possibility that VDAC1 oligomerization may act upstream of, or cooperate with, BAX/BAK oligomerization during mitochondrial damage. Therefore, in our study, the inhibitory effects of both VBIT-4 and MSN-50 on cytosolic mtDNA accumulation suggest that heat stress-induced mtDNA leakage involves coordinated mitochondrial membrane permeabilization, and that BAX/BAK oligomerization may act as the dominant pathway while VDAC1 oligomerization functions as a contributing regulatory event. However, more in-depth studies are warranted regarding the mechanisms of mtDNA leakage under heat stress.

Collectively, leptin effectively prevented cytosolic mtDNA leakage by inhibiting aberrant mPTP opening, and suppressing oligomerization of BAX/BAK and/or oligomerization of VDAC1.

### Leptin suppresses activation of the cGAS-STING pathway

This study found that heat stress increased levels of cGAS and STING, which was consistent with observations in spermatogonia and macrophages [36, 37]. Notably, leptin treatment effectively attenuated the heat stress-induced activation of cGAS and STING in Sertoli cells, as reported in high glucose-treated cardiomyocytes [20]. These findings suggest that leptin possesses a conserved capacity to suppress cGAS-STING activation across diverse cell types and stress conditions.

Given that TBK1-IRF3 phosphorylation represents a canonical downstream event following cGAS-STING activation, we subsequently assessed this signaling axis. We found that heat stress triggered the phosphorylation of both TBK1 and IRF3 in Sertoli cells, aligning with the previous report in macrophages [36]. In macrophages, various stress factors, including ethylene glycol-derived advanced glycation end products (AGEs) [38], peptidyl-prolyl isomerase A [39], and lipopolysaccharides [38], activated STING signaling. Furthermore, Tan et al. [40] reported that tetrandrine induced STING‑dependent TBK1 and IRF3 phosphorylation in CD8⁺ T cells. Collectively, these observations indicate that STING signaling is a widespread response to multiple stressors, leading to the activation of the TBK1-IRF3 axis.

Additionally, the cGAS-STING pathway is known to activate the NF-κB signaling cascade. In the current study, we found that heat stress induced IκBα phosphorylation, which aligned with pathological changes observed in testicular tissue and endothelial cells [41–43]. Remarkably, leptin treatment inhibited IκBα phosphorylation. Phosphorylation and subsequent degradation of IκBα promote the release and nuclear translocation of p65 [44, 45]. In the current study, our results demonstrate that heat stress significantly increased the nuclear localization of p65 and elevated the levels of pro-inflammatory cytokines (IL-6 and IL-1α), aligning with previous findings in testicular tissue, endothelial cells, and microglia [41–43, 46]. The upregulation of these cytokines may further amplify the inflammatory response through synergistic mechanisms [47–49]. Notably, leptin treatment effectively reduced p65 nuclear accumulation and suppressed the production of pro-inflammatory cytokines, which was consistent with previous findings in LPS-induced astrocytes [21]. Furthermore, following inhibition of cGAS or STING, leptin no longer reduced the upstream signaling molecules (p‑TBK1, p‑IRF3, p‑IκBα, p65) but continued to lower IL‑1α and IL‑6. These results support that leptin regulates these cytokines in part through the cGAS‑STING pathway. In support of this notion, leptin has been shown to suppress p38 MAPK signaling and reduce IL‑6 levels in astrocytes and hepatocytes [21, 50]. Alternatively, leptin may influence the post‑transcriptional stability of these cytokine mRNAs. Elucidating these mechanisms warrants future study.

Together, leptin effectively suppresses heat stress-induced cGAS-STING activation, thereby inhibiting downstream signaling cascades. Moreover, leptin reduces the secretion of pro‑inflammatory cytokines at least partially through a cGAS‑STING‑independent mechanism in Sertoli cells.

### Leptin alleviates heat stress-induced mitoxyperilysis in Sertoli cells

In the present study, we observed that heat stress significantly elevated mtROS levels and promoted physical contact between mitochondria and the plasma membrane, leading to membrane rupture and the initiation of mitoxyperilysis. Notably, leptin treatment effectively alleviated this phenotype. Wang et al. [17] reported that macrophages exhibited mitoxyperilysis under the condition of lipopolysaccharide stimulation combined with carbon starvation, the novel cell death driven by a burst of mtROS and sustained mitochondria-plasma membrane contact. They found that knocking out *Bax*/*Bak* significantly reduced the cell death [17], suggesting that *Bax* and *Bak* may be involved in the regulation of mitoxyperilysis [17]. However, gene knockout approaches preclude direct observation of Bax/Bak oligomerization.

The current study revealed that heat stress significantly increased BAX/BAK oligomerization. It is well established that BAX and BAK oligomerize to form macropores in the outer mitochondrial membrane, facilitating mtDNA leakage into the cytosol [10, 27, 31]. The leaked mtDNA subsequently activates cGAS-STING signaling, which in turn drives the release of pro-inflammatory cytokines, including IL-6 and IL-1α [14, 29, 51–53].

Notably, these inflammatory factors can further exacerbate oxidative stress, creating a detrimental feedback loop. For instance, IL-6 has been shown to induce a significant increase in mtROS levels by activating JAK/STAT signaling in vascular endothelial and skeletal muscle cells [54, 55]. Similarly, Li et al. [56] reported that IL-6 promoted ROS production by upregulating p47phox in neutrophils. Moreover, Zell et al. [57] found that IL-1α directly inhibited pyruvate dehydrogenase activity within the mitochondrial respiratory chain of cardiomyocytes, leading to impaired oxidative phosphorylation. Such dysfunctional electron transport chain complexes often undergo electron leakage, generating large amounts of superoxide anions and further intensifying intracellular oxidative stress [58].

Importantly, this inflammation‑induced mitochondrial dysfunction may in turn promote further mtDNA release into the cytosol, thereby reactivating the cGAS‑STING pathway and driving additional production of pro‑inflammatory cytokines, thus creating a self‑amplifying positive feedback loop. In support of this notion, IL‑6 has been shown to induce mtDNA leakage and subsequent cGAS‑STING activation in endometrial carcinoma cells [59]. Similarly, TNF triggers mtDNA release and cGAS‑STING‑dependent interferon responses [60], and exogenous IL‑1β directly activates cGAS‑STING‑dependent IRF3 signaling in multiple cell types [29]. Moreover, mitochondrial stress has been reported to release both mtDNA and ROS, activating cGAS‑STING and perpetuating chronic inflammation [61, 62]. Our finding that leptin both suppresses the initial activation of cGAS‑STING and reduces the levels of IL‑6 and IL‑1α suggests that leptin may interrupt this vicious cycle, thereby contributing to the mitigation of mitoxyperilysis.

Thus, this study provided the first evidence that heat stress triggered mtDNA leakage through BAX/BAK oligomerization, thereby activating cGAS-STING signaling, driving inflammatory cytokine release, and culminating in mitoxyperilysis. Remarkably, leptin inhibited the BAX/BAK oligomerization.

### Leptin maintains metabolic homeostasis

In the present study, heat stress significantly reduced the mitochondrial membrane potential (ΔΨm) and ATP levels, which were consistent with previous findings in porcine, goat and bovine Sertoli cells [8, 63, 64]. Leptin treatment effectively alleviated the heat stress-induced decreases in ΔΨm and ATP levels and attenuated the reduction in citrate synthase activity, which were in line with previous studies in adipocytes and breast cancer cells by leptin treatment [65, 66]. Moreover, we found that heat stress significantly increased the activity of pyruvate kinase, a key rate-limiting enzyme in glycolysis, leading to lactate accumulation. Leptin reduced this lactate accumulation, similarly to its effect in breast cancer cells [65]. Elevated lactate levels and enhanced glycolysis contribute to ROS accumulation and inflammatory responses in breast cancer cells and neutrophils [67, 68]. Notably, lactate is also a major metabolic substrate supplied by Sertoli cells to developing germ cells and is essential for germ cell survival under physiological conditions [69, 70]. Therefore, the excessive lactate accumulation observed under heat stress may reflect metabolic dysregulation rather than a beneficial metabolic adaptation. By reducing lactate accumulation, leptin may alleviate oxidative stress and help maintain a stable metabolic microenvironment that supports germ cell survival.

### Study limitations

A limitation of our current study, however, is the lack of genetic gain- and loss-of-function approaches which will be helpful for advancing our understanding of how mitochondrial pores modulate mtDNA release. Meanwhile, our findings were obtained exclusively in primary porcine Sertoli cells under acute heat stress in vitro, whether the same protective mechanism of leptin operates under chronic heat stress or in vivo remains to be investigated.

## Conclusion

In summary, heat stress compromises mitochondrial integrity and function in Sertoli cells by facilitating pathological mPTP opening and oligomerization of BAX/BAK and VDAC1. This cascade triggers mtDNA leakage and subsequent cGAS-STING activation, which fuels inflammatory signaling and ultimately culminates in mitoxyperilysis. Leptin effectively attenuates this deleterious sequence by preserving mitochondrial membrane integrity. These findings suggest that leptin attenuates heat stress-induced mitoxyperilysis by suppressing mtDNA-cGAS-STING signaling in porcine Sertoli cells. This study highlights its potential to support future therapeutic strategies for heat stress-related male reproductive dysfunction.

## Supplementary Information

Additional file 1: Table S1. Information on antibodies used in Western blot and immunofluorescence experiments. Table S2. List of primers used for qPCR analysis in this study.

Additional file 2: Text S1. Sertoli cell collection. Text S2. Mitochondrial function assays. Text S3. Mitochondrial DNA stress response assay. Fig. S1. Purity analysis of primary Sertoli cells. Fig. S2. Inhibition of BAX/BAK or VDAC1 oligomerization blocks heat stress-induced mtDNA release and subsequent cGAS-STING pathway activation. Fig. S3. Leptin inhibits heat stress-induced p65 activation in Sertoli cells.

Additional file 3: The full uncropped Gels and Blots images.

## Data Availability

All data generated or analysed during this study are included in this published article and its supplementary information files.

## Abbreviations

BAK: BCL-2 homologous antagonist killer
BAX: BCL-2-associated X protein
cGAS: Cyclic GMP-AMP synthase
ELISA: Enzyme-linked immunosorbent assay
IL-1α: Interleukin 1 alpha
IL-6: Interleukin 6
IRF3: Interferon regulatory factor 3
mPTP: Mitochondrial permeability transition pore
mtDNA: Mitochondrial DNA
mtROS: Mitochondrial reactive oxygen species
NF-κB: Nuclear factor kappa-light-chain-enhancer of activated B cells
PI: Propidium iodide
ROS: Reactive oxygen species
STING: Stimulator of interferon genes
TBK1: TANK-binding kinase 1
VDAC1: Voltage-dependent anion channel 1
