# Supplementary material for: Leptin attenuates heat stress-induced mitoxyperilysis in porcine Sertoli cells by suppressing the mtDNA-cGAS-STING axis
Source: J Anim Sci Biotechnol. 2026 Sep 6;17:172. doi: 10.1186/s40104-026-01489-6 (PMC13546511; doi:10.1186/s40104-026-01489-6)
Supplement: Supplementary file 1 [file 40104_2026_1489_MOESM1_ESM.docx]

**Supporting Information**

Leptin attenuates heat stress-induced mitoxyperilysis in porcine Sertoli cells by suppressing the mtDNA-cGAS-STING axis

Ming Guo ^1^, Jinyang Zhang ^2^, Shanshan Gu ^2^, Yawen Gao ^2^, Zhendong Zhu ^3^, Tao Zhang ^2^, Hongzhao Lu ^2^, Jun Luo ^1^*, Wenxian Zeng ^1,2^*

^1^College of Animal Science and Technology, Northwest A&F University, Yangling, Shaanxi 712100, China.

^2^School of Biological Science and Engineering, Shaanxi University of Technology, Hanzhong, Shaanxi 723001, China

^3^College of Animal Science and Technology, Qingdao Agricultural University, Qingdao 266109, China

**^*^Corresponding authors:** Jun Luo (E-mail: luojun@nwsuaf.edu.cn; Wenxian Zeng (E-mail: zengwenxian2015@126.com)

**Corresponding email:** zengwenxian2015@126.com;

**Table S1. Information on antibodies used in Western blot and immunofluorescence experiments.**

| **Antibody** | **Species Source** | **Supplier** | **Identifier** | **Dilution** | |
| --- | --- | --- | --- | --- | --- |
|  |  |  |  | **WB** | **IF** |
| VDAC1 | Rabbit | Proteintech | 81538-1-RR | 1:2000 |  |
| BAK | Rabbit | Proteintech | 29552-1-AP | 1:2000 |  |
| BAX | Rabbit | BBI | D120073-0010 | 1:2000 |  |
| cGAS | Rabbit | Huaan Biotechnology | HA500023 | 1:2000 |  |
| p-STING | Rabbit | abmart | TA7416 | 1:1000 |  |
| STING | Rabbit | Proteintech | 19851-1-AP | 1:2000 |  |
| TBK1 | Mouse | Proteintech | 67211-1-Ig | 1:2000 |  |
| p-TBK1 | Rabbit | abcam | Ab109272 | 1:2000 |  |
| IRF3 | Rabbit | Proteintech | 11312-1-AP | 1:2000 |  |
| p-IRF3 | Rabbit | Proteintech | 29528-1-AP | 1:1500 |  |
| IκBα | Rabbit | Proteintech | 10268-1-AP | 1:2000 |  |
| p-IκBα | Mouse | Thermo Fisher  Scientific | MA5-15224 | 1:2000 |  |
| p65 | Mouse | Proteintech | 66535-1-Ig | 1:2000 |  |
| SOX9 | Rabbit | abcam | Ab185966 |  | 1:200 |
| β-actin | Rabbit | Biodragon | B2243 | 1:4000 |  |
| Goat anti-rabbit IgG, HRP-conjugated | Goat | CWBIO | CW0103S | 1:5000 |  |
| Goat anti-mouse IgG, HRP-conjugated | Goat | CWBIO | CW0102S | 1:5000 |  |
| Alexa Fluor 594 donkey anti-rabbit IgG (H+L) | Donkey | YEASEN | 34206ES60 | 1:400 |  |

**Table S2. List of primers used for qPCR analysis in this study.**

| **Gene** | **Sense Primer (5’ to 3’)** | **Anti-sense Primer (5’ to 3’)** |
| --- | --- | --- |
| *mt-COX1* | GGAGCGGGTACTGGATGAAC | CACCTGCAAGGTGTAGGGAG |
| *mt-ND1* | CGAAAAGGTCCAAATGTCGT | CTGAAGATGTAGCGGGTCGT |
| *D-loop1* | CGAAACCAGACGAGCTACCC | CGGACAACCAGCTATCACCA |
| *D-loop2* | GAAACCAGACGAGCTACCCA | CACCAGGCTCGTTAGGCTTT |
| *D-loop3* | GATACCCCGAAACCAGACGA | TCGGACAACCAGCTATCACC |
| *IL-6* | AAGCTGCAGTCACAGAACGA | TGGACGGCATCAATCTCAGG |
| *IL-1α* | ATGCCAGCTATGAGCCACTTCCT | CTGCCATCACCACACTGTCCTTG |
| *β-actin* | AGATCAAGATCATCGCGCCT | ATGCAACTAACAGTCCGCCT |
