# Supplementary material for: Leptin attenuates heat stress-induced mitoxyperilysis in porcine Sertoli cells by suppressing the mtDNA-cGAS-STING axis
Source: J Anim Sci Biotechnol. 2026 Sep 6;17:172. doi: 10.1186/s40104-026-01489-6 (PMC13546511; doi:10.1186/s40104-026-01489-6)
Supplement: Supplementary file 2 [file 40104_2026_1489_MOESM2_ESM.docx]

**Supporting Information**

Leptin attenuates heat stress-induced mitoxyperilysis in porcine Sertoli cells by suppressing the mtDNA-cGAS-STING axis

Ming Guo ^1^, Jinyang Zhang ^2^, Shanshan Gu ^2^, Yawen Gao ^2^, Zhendong Zhu ^3^, Tao Zhang ^2^, Hongzhao Lu ^2^, Jun Luo ^1^*, Wenxian Zeng ^1,2^*

^1^College of Animal Science and Technology, Northwest A&F University, Yangling, Shaanxi 712100, China.

^2^School of Biological Science and Engineering, Shaanxi University of Technology, Hanzhong, Shaanxi 723001, China

^3^College of Animal Science and Technology, Qingdao Agricultural University, Qingdao 266109, China

**^*^Corresponding authors:** Jun Luo (E-mail: luojun@nwsuaf.edu.cn); Wenxian Zeng (E-mail: zengwenxian2015@126.com)

**Corresponding email:** zengwenxian2015@126.com;

**Text S1. Sertoli cell collection**

Sertoli cells were isolated from the testes of 7-day-old Landrace piglets according to a previously described protocol. The isolation procedure was conducted as follows:

Immediately after collection, testicular tissues were immersed in ice-cold phosphate-buffered saline (PBS) and transported to the laboratory under sterile conditions. Under a laminar flow hood, the tunica albuginea was carefully removed, and the testicular parenchyma was minced into ~2 mm³ fragments. To dissociate seminiferous tubules, the tissue fragments were incubated with collagenase type IV (2 mg/mL, Gibco) at 37°C for 20 min with gentle agitation. The resulting seminiferous tubules were subsequently digested with 0.25% trypsin-EDTA (Cellmax, CPT101.02) under the same conditions for an additional 20 min to obtain a single-cell suspension. Sertoli cells were enriched by differential adhesion. The purified cells were then cultured in Dulbecco’s Modified Eagle Medium (DMEM) supplemented with 10% fetal bovine serum and 1% penicillin-streptomycin in a humidified incubator at 37°C with 5% CO₂ until further use.

All culture conditions, including medium composition, cell seeding density, and CO₂ concentration, were maintained consistently across experimental groups to ensure comparability of results.

**Text S2. Mitochondrial function assays**

**Hexokinase** activity was measured using a **Hexokinase** Activity Assay Kit (**Solarbio, China**), according to the manufacturer’s instructions. Briefly, cells were homogenized in ice-cold assay buffer and centrifuged at 8,000 × g for 10 min at 4 °C. The supernatant was collected for analysis. The reaction mixture was prepared by combining the components in equal proportions, and the absorbance at 340 nm was measured immediately. The mixture was then incubated at 37°C for 5 min, followed by a second absorbance measurement. Hexokinase activity was normalized to total protein concentration and expressed as U/mg protein.

Phosphofructokinase activity was measured using a Phosphofructokinase Activity Assay Kit (Solarbio, China), following the manufacturer’s instructions. Cells were homogenized in ice-cold assay buffer and centrifuged at 10,000 × g for 10 min at 4°C, and the supernatant was collected for analysis. The reaction mixture was incubated at 37°C for 30 min, after which a color-developing reagent was added. Absorbance was measured at 550 nm using a microplate reader (BioTek, USA). Phosphofructokinase activity was normalized to total protein concentration and expressed as U/mg protein.

Pyruvate kinase M activity was measured using a Pyruvate Kinase M Activity Assay Kit (Beyotime, China) according to the manufacturer’s instructions. Briefly, cell lysates were mixed with the Amplex Red reaction working solution and incubated in the dark at 37°C for 20 min. Fluorescence intensity was measured and normalized to protein concentration.

Lactate dehydrogenase activity was measured using a Total Lactate Dehydrogenase Activity Kit (Beyotime, China). Briefly, cell lysates were mixed with WST-8 working solution and chromogenic reagent and incubated at 37°C for 30 min. Absorbance was measured at 530 nm at 0 min and 20 min. Lactate dehydrogenase activity was normalized to protein concentration.

Lactic acid content was measured using a Lactic Acid Assay Kit (Nanjing Jiancheng, China) according to the manufacturer’s instructions. Briefly, cell samples were mixed with enzyme working solution and chromogenic reagent, incubated at 37°C for 10 min, and the reaction was terminated by adding a stop solution. Absorbance was measured at 530 nm.

Pyruvic acid content was determined using a Pyruvic Acid Assay Kit (Beyotime, China) according to the manufacturer’s instructions. Cell lysates were mixed with the Amplex Red reaction working solution and incubated in the dark at 37°C for 30 min. Fluorescence intensity was measured and normalized to protein concentration.

**Text S3. Mitochondrial DNA stress response assay**

Cytosolic mtDNA was isolated using a digitonin-based subcellular fractionation method with minor modifications. Briefly, cells were resuspended in 500 μL lysis buffer (50 mmol/L NaCl, 50 mmol/L HEPES, 20 μg/mL digitonin) and gently rotated for 10 min at room temperature to selectively permeabilize the plasma membrane. Following centrifugation at 1,000 × g for 5 min to remove intact cells and organelles, the supernatant was subjected to a second centrifugation at 17,000 × g for 25 min to pellet residual debris. Cytosolic mtDNA was then extracted from the resulting supernatant using the TIANamp Genomic DNA Kit (TIANGEN, China), according to the manufacturer’s instructions.

The abundance of mtDNA in the cytosolic fraction was quantified by absolute qPCR using mtDNA-specific primers targeting the D-loop region (D-loop 1, D-loop 2, D-loop 3; primer sequences are listed in Table S2).

In parallel, cells were co-stained with MitoTracker Red (Cell Signaling Technology, USA) and MitoDNA Green 530 (AAT Bioquest, USA) to visualize the spatial distribution of mtDNA relative to mitochondrial structures. Fluorescence images were acquired using a fluorescence microscope, and mtDNA release was assessed based on the degree of co-localization between mitochondrial and mtDNA signals.

**Fig. S1** Identification of Sertoli cell purity and screening of the optimal leptin concentration. (A) Immunocytochemistry analysis and quantification of SOX9^+^ expression in Sertoli cells. Scale bar = 50 μm. (B) Cell viability of Sertoli cells treated with different concentrations of leptin (0, 3, 6, 9, 12 nmol/L), presented as the percentage relative to the control group (0 nmol/L). Data are presented as the mean ± SD of at least three independent experiments (n ≥ 3). Different lowercase letters above the bars indicate significant differences among groups (*P* < 0.05).

**Fig. S2** Inhibition of BAX/BAK or VDAC1 oligomerization blocks heat stress-induced mtDNA release and subsequent cGAS-STING pathway activation. (**A-C**) Quantitative qPCR analysis showing fold changes of three mtDNA D-loop fragments (D-loop 1, D-loop 2, D-loop 3) in different groups. (**D-F**) Western blot detection and densitometric quantification of cGAS (**D**), p-STING (**E**), and STING (**F**) levels in Sertoli cells; β-actin was used as the loading control.

**Fig. S3** Leptin inhibits heat stress-induced p65 activation in Sertoli cells. (A) Western blot analysis showing the relative protein levels of p65 in different groups. β-actin was used as the internal loading control. (B) Immunofluorescence staining of p65 (red) counterstained with DAPI (blue) to visualize nuclei in each group. Scale bar = 50 μm. Data are presented as the mean ± SD of at least three independent experiments (n ≥ 3). Different lowercase letters above the bars indicate significant differences among groups (*P* < 0.05).
