# Supplementary material for: Leptin attenuates heat stress-induced mitoxyperilysis in porcine Sertoli cells by suppressing the mtDNA-cGAS-STING axis
Source: J Anim Sci Biotechnol. 2026 Sep 6;17:172. doi: 10.1186/s40104-026-01489-6 (PMC13546511; doi:10.1186/s40104-026-01489-6)
Supplement: Supplementary file 3 [file 40104_2026_1489_MOESM3_ESM.docx]

**Supporting Information**

Leptin attenuates heat stress-induced mitoxyperilysis in porcine Sertoli cells by suppressing the mtDNA-cGAS-STING axis

Ming Guo ^1^, Jinyang Zhang ^2^, Shanshan Gu ^2^, Yawen Gao ^2^, Zhendong Zhu ^3^, Tao Zhang ^2^, Hongzhao Lu ^2^, Jun Luo ^1^*, Wenxian Zeng ^1,2^*

^1^College of Animal Science and Technology, Northwest A&F University, Yangling, Shaanxi 712100, China.

^2^School of Biological Science and Engineering, Shaanxi University of Technology, Hanzhong, Shaanxi 723001, China

^3^College of Animal Science and Technology, Qingdao Agricultural University, Qingdao 266109, China

**^*^Corresponding authors:** Jun Luo (E-mail: [luojun@nwsuaf.edu.cn](mailto:luojun@nwsuaf.edu.cn)); Wenxian Zeng (E-mail: zengwenxian2015@126.com)

**Corresponding email:** zengwenxian2015@126.com;

1. Original Western blot data for Fig. 2C in the main text. Numbers on the left of each subpanel represent protein molecular weight (kDa);

3. Original Western blot data for Fig. 2E in the main text. Numbers on the left of each subpanel represent protein molecular weight (kDa);

4. Original Western blot data for Fig. 2G in the main text. Numbers on the left of each subpanel represent protein molecular weight (kDa);

5. Original Western blot data for Fig. 3 in the main text. Numbers on the left of each subpanel represent protein molecular weight (kDa);

6. Original Western blot data for Fig.S2 in the main text. Numbers on the left of each subpanel represent protein molecular weight (kDa);

6. Original immunofluorescence image for Fig. 2I.

7. Original immunofluorescence image for Fig. 5C.

8. Original immunofluorescence image for Fig. S1A.

9. Original immunofluorescence image for Fig. S3.
